# Objectively Measured Baseline Physical Activity Patterns in Women in the mPED Trial: Cluster Analysis

**DOI:** 10.2196/publichealth.9138

**Published:** 2018-02-01

**Authors:** Yoshimi Fukuoka, Mo Zhou, Eric Vittinghoff, William Haskell, Ken Goldberg, Anil Aswani

**Affiliations:** ^1^ Department of Physiological Nursing/Institute for Health & Aging University of California, San Francisco San Francisco, CA United States; ^2^ Department of Industrial Engineering and Operations Research University of California, Berkeley Berkeley, CA United States; ^3^ Department of Epidemiology & Biostatistics University of California, San Francisco San Francisco, CA United States; ^4^ Stanford Prevention Research Center Stanford University Palo Alto, CA United States; ^5^ Department of Electrical Engineering and Computer Sciences University of California, Berkeley Berkeley, CA United States

**Keywords:** accelerometer, physical activity, cluster analysis, women, randomized controlled trial, machine learning, body mass index, metabolism, primary prevention, mHealth

## Abstract

**Background:**

Determining patterns of physical activity throughout the day could assist in developing more personalized interventions or physical activity guidelines in general and, in particular, for women who are less likely to be physically active than men.

**Objective:**

The aims of this report are to identify clusters of women based on accelerometer-measured baseline raw metabolic equivalent of task (MET) values and a normalized version of the METs ≥3 data, and to compare sociodemographic and cardiometabolic risks among these identified clusters.

**Methods:**

A total of 215 women who were enrolled in the Mobile Phone Based Physical Activity Education (mPED) trial and wore an accelerometer for at least 8 hours per day for the 7 days prior to the randomization visit were analyzed. The k-means clustering method and the Lloyd algorithm were used on the data. We used the elbow method to choose the number of clusters, looking at the percentage of variance explained as a function of the number of clusters.

**Results:**

The results of the k-means cluster analyses of raw METs revealed three different clusters. The unengaged group (n=102) had the highest depressive symptoms score compared with the afternoon engaged (n=65) and morning engaged (n=48) groups (overall *P<*.001). Based on a normalized version of the METs ≥3 data, the moderate-to-vigorous physical activity (MVPA) evening peak group (n=108) had a higher body mass index (*P*=.03), waist circumference (*P*=.02), and hip circumference (*P*=.03) than the MVPA noon peak group (n=61).

**Conclusions:**

Categorizing physically inactive individuals into more specific activity patterns could aid in creating timing, frequency, duration, and intensity of physical activity interventions for women. Further research is needed to confirm these cluster groups using a large national dataset.

**Trial Registration:**

ClinicalTrials.gov NCT01280812; https://clinicaltrials.gov/ct2/show/NCT01280812 (Archived by WebCite at http://www.webcitation.org/6vVyLzwft)

## Introduction

### Background

Increasing physical activity is associated with a reduction in chronic illnesses and an increase in psychological well-being [1-3]. The 2008 Physical Activity Guidelines for Americans recommends US adults engage in a total 150 minutes of moderate-intensity aerobic activity (ie, brisk walking) each week or 75 minutes of vigorous-intensity aerobic activity each week, to be done in at least 10-minute bouts of activity [4]. The guidelines were developed mainly based on self-reported physical activity data in relation to health outcomes [5]. Since the current guidelines were issued, more objectively measured physical activity data in relation to health outcomes have become available. Recently, the US Department of Health and Human Services announced that they intend to publish new physical activity guidelines in 2018 [6].

Recent investigations have shown that there is a large discrepancy between self-reported and objectively measured moderate-to-vigorous physical activity (MVPA) [7]. Although half of adults meet the current physical activity guidelines by self-report, only 3.5% of American adults meet these guidelines by accelerometer [8]. In particular, women and older adults are less likely to be physically active than men and younger adults regardless of measurement methods [9,10]. Dichotomization of meeting or not meeting the physical activity guidelines provides only one-dimensional information. However, identifying patterns of physical activity throughout the day may help develop more personalized interventions or physical activity guidelines in general, and in particular for women and older adults.

Cluster analysis is a useful statistical technique that can allocate observations/individuals into groups based on similar characteristics [11]. In the past, a cluster analysis technique was used to cluster individuals based on self-reported physical activity and sedentary behavior. Few studies utilized objectively measured (ie, accelerometer) physical activity data. In a large cohort study in Hong Kong, two clusters were identified: (1) the active group characterized by a routine activity pattern on weekdays and a varied pattern on weekends and (2) the less active group characterized by a low activity pattern on weekdays and weekends [12]. A total of 72% of adults in this Hong Kong sample were classified as the less active group, and the daily average duration of MVPA in the active groups was two times greater than in the less active group. One of the limitations of this cohort study was that only four consecutive days of accelerometer data were used.

### Goals of This Study

Our research team had a unique opportunity to analyze seven consecutive days of accelerometer data in women who were screened and completed the run-in period of the Mobile Phone Based Physical Activity Education (mPED) randomized controlled trial (RCT). To our knowledge, no study has used cluster analyses to explore daily patterns of physical activity using seven consecutive days of accelerometer data in female adults. The aims of this paper are (1) to identify clusters of women who enrolled in the mPED study based on overall accelerometer-measured baseline physical activity and MVPA and (2) to compare sociodemographic and cardiometabolic risks among these identified clusters.

## Methods

### Study Design and Sample

The mPED study is a RCT of the app-based physical activity intervention in physically inactive women (trial registration: ClinicalTrials.gov NCT01280812). Detailed descriptions of the study design and study protocol have been published previously [7,13,14]. The study protocol was approved by the University of California, San Francisco Committee on Human Research and the Data and Safety Monitoring Board. All participants provided written consent prior to study enrollment. In this paper, we analyzed only the sociodemographic, clinical, and self-reported questionnaires data collected at the screening/baseline study visit and accelerometry data collected during the last 7 days of the run-in period prior to a randomization visit.

Initial inclusion criteria for the mPED trial were (1) physically inactive at work and/or during leisure time based on the Stanford Brief Activity Survey [15], (2) intent to be physically active, (3) female aged 25 to 69 years, (4) access to a home telephone or mobile phone, (5) speak and read English, (6) body mass index (BMI) of 18.5 to 43.0 kg/m^2^, and (7) no mild cognitive impairment screened by the Mini-Cog test [16,17]. Initial exclusion criteria were (1) known medical conditions or physical problems that require special attention in an exercise program, (2) planning an international trip during the next 4 months (which could interfere with daily server uploads of mobile phone data), (3) pregnant/gave birth during the past 6 months, (4) severe hearing or speech problem, (5) history of eating disorder, (6) current substance abuse, (7) current participation in lifestyle modification programs or research studies that may confound study results, and (8) history of bariatric surgery or plans for bariatric surgery in the next 12 months.

In total, 318 women came in for a screening/baseline visit. Of those, 57 did not start or complete the run-in period and 46 did not have sufficient accelerometer wear time of at least 8 hours per day for the last 7 days prior to the randomization visit. The remaining 215 participants were analyzed in this report.

### Measures

A triaxial accelerometer (HJA-350IT, Active Style Pro, Omron Healthcare Co, Ltd) was used to assess objectively measured physical activity [18,19]. Its dimensions are 74×46×34 mm (width/height/depth) including the clip, and it weighs 60 grams (2.1 oz). Throughout the run-in period, participants were asked to wear the accelerometer all day on their waist, except when showering, bathing, swimming, or sleeping, from the time they got up in the morning until they went to bed at night. All participants were also instructed to engage in their regular daily activity and not increase this activity during the run-in period. The accelerometer displayed only date and time. To avoid providing any feedback and to collect the clean baseline activity data, neither the step counts nor metabolic equivalent of task (MET) values were displayed. Activity data were stored minute by minute for the entire duration of the run-in period, and the accelerometer’s data was automatically reset at midnight. A trained research staff downloaded the data to a personal computer with the software program provided by the manufacturer in the research office prior to randomization visit. In this paper, only recorded accelerometer data during the seven consecutive days prior to the randomization visit were used to identify patterns of physical activity. In order for accelerometer data to be valid, all 7 days of accelerometer activity needed to indicate at least 8 hours per day of recorded wear time for the device. The METs determined by this accelerometer are closely correlated with METs calculated using energy expenditure measured by indirect calorimetry [20,21]. This accelerometer was programed to collect physical activity intensity every 10 seconds per minute and the mean intensity value of a 1-minute epoch was calculated as the mean of six 10-second epochs. Moderate- or vigorous-intensity activity was defined as ≥3 to <6 or ≥6 METs, respectively, using the Compendium of Physical Activities [20,21].

The Center for Epidemiological Studies Depression Scale (CES-D) is a 20-item questionnaire widely used for assessing symptoms of depression [22]. Scores can range from 0 to 60, with higher scores indicating more depressive symptoms. The 12-item Short-Form Health Survey (SF-12) is an instrument derived from the longer 36-item Short-Form Survey, which was designed to measure general health functioning [23]. The SF-12 provides two summary scores, the Physical Component Summary and the Mental Component Summary. Scores are standardized; the mean score in the population is 50 with a standard deviation of 10 points. Higher scores indicate better functioning in physical function or mental status. The Television/Computer Usage Scale is a semistructured interview that estimates an individual’s time spent (1) using a computer, Internet, or mobile phone and (2) watching television or movies for the 7 days prior to the interview. This measure was developed by the investigator prior to the trial. A trained research staff used the 7-day worksheet to assess the duration of these activities for the 7 days. The Social Support for Exercise Survey consists of 13 items assessing the level of perceived support from family and friends for behavior changes related to exercise [24]. Each item is scored separately for family and friends, and scores can range from 13 to 65 with higher scores indicating greater support. The Barriers to Being Active Quiz consists of 21 items assessing seven subscales: lack of time, lack of social influence, lack of energy, lack of willpower, fear of injury, lack of skill, and lack of resources. Each subscale can range from 0 to 9 and total scores can range from 0 to 63, with higher scores indicating more barriers to physical activity [25]. The Modified Self-Efficacy for Physical Activity Scale, consisting of six items (five original questions plus one extra question), was used to assess confidence in one’s ability to exercise, an important determinant of the stages of change for exercise behavior. Total scores can range from 6 to 30, with higher scores indicating greater self-efficacy for physical activity. Anthropometric measures included height, weight in kilograms, and waist and hip circumferences; BMI was calculated based on height and weight in kilograms at the screening/baseline visit. Participants were asked to change to a hospital gown and remove their shoes prior to the measurement.

### Statistical Analysis

The k-means clustering method (hereafter referred to as k-means) [26] was applied to the accelerometer dataset. This method takes as input: (1) a set of data points with each data point corresponding to a single individual, (2) a subset of characteristics summarizing each data point, and (3) a number of desired clusters. In the terminology of machine learning, the subset of summarizing characteristics is known as the features of the data [27]. As output, this method separates the data points into distinct groups (ie, clusters) such that the data points within each group have similar characteristics and the data points between different groups have different characteristics.

To apply k-means, we used the Lloyd algorithm [28] to perform the computations. To ensure accurate modeling, we repeated the Lloyd algorithm a total of 25 times with random initialization to find the most accurate clustering (as measured by the percentage of variance of the data explained by the identified cluster medians). To determine an appropriate number of desired clusters, we applied the elbow method [29]. The elbow method selects the number of clusters to be such that adding an additional cluster does not significantly reduce the within-group sum of squares. We applied k-means two times, and each time we used a different subset of summarizing characteristics. The two different subsets we used in our analysis are described subsequently. After applying k-means, chi-square or ANOVA tests were used to compare sociodemographic and clinical characteristics among these clustered groups. To visualize the clusters, we first computed the mean for each group selected by k-means of the corresponding data points. Then we applied Loess smoothing [30] in time to better visualize average temporal trends. Statistical analyses were performed in R 3.1.1 [31].

### Raw METs Data

The k-means clustering was applied to raw METs data from each enrolled participant to evaluate if raw minute-level METs were able to classify participants by physical activity and time to do physical activity. All observations including day and night were included because participants engaged in activity at various time points. Thus, naively removing night data would lead to a loss of information. Specifically, the features for each individual consisted of a 10,080-dimensional vector comprised of consecutive (at the minute interval) METs observations for 7 days. Missing data occurred mainly during nighttime and hence were simply replaced by 1, which is the METs reading for a stationary individual.

**Figure 1:**
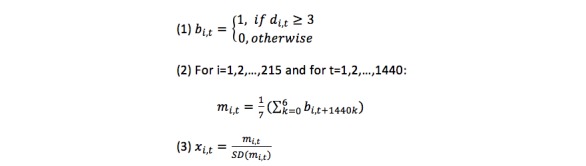
Equations 1-3.

### Normalized METs ≥3 Data

We also explored how MVPA (METs≥3) were associated with sociodemographic data and clinical outcomes. Thus, k-means clustering was applied to a normalized version of the METs data from each enrolled participant, and the data were normalized as follows: suppose for each participant *i* (i=1,2,...,215) and time *t* (t=1,2,...,10,080), the raw METs record was *d*_i,t_. We first converted the raw METs records into binary values (see Equation 1 in Figure 1). This binary conversion corresponds to whether the participant was having MVPA or not, which is an important indicator to characterize a person’s physical activity level. Next, we averaged the binary values for the 7 days to compute the MVPA frequency for a typical day for each individual. We ended up with 1440 features for each individual, which indicates the minute-level normalized METs for the day averaged over all days (see Equation 2 in Figure 1).

Finally, we normalized this vector for each individual by time to have unit Euclidean norm (see Equation 3 in Figure 1). This normalization ensured that the overall physical activity level of each participant was similar and that the clustering results then categorized participants using the time in day (ie, morning, noon, evening) information.

## Results

### Overall Participants’ Characteristics

Overall, the mean age of participants was 52.4 (SD 11.2) years, 54.4% (117/215) were white, 48.8% (105/215) were single or divorced, and 73.0% (157/215) were well educated, reporting college- or graduate-level educations. In addition, 49.3% (106/215) had used a pedometer and 57.2% (123/215) had participated in a diet/weight loss plan prior to study enrollment. The majority of the sample (80.5%, 173/215) drove a car at least once per week.

### Clustering on Raw METs Data

**Figure 2:**
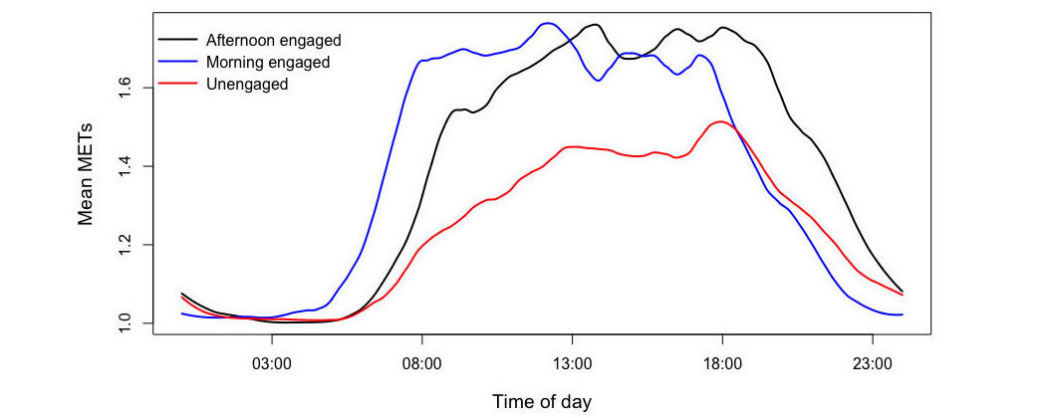
A k-means cluster analysis of raw METs (metabolic equivalent of tasks) data with the Lloyd algorithm (N=215) for physical activity frequency during the day for each cluster.

The k-mean clustering separated the participants into three groups (Figure 2). The elbow method indicated that separating the data into four groups did not reduce within-group sum of squares significantly. Therefore, we chose three clusters for this analysis (Multimedia Appendix 1). There were 65, 48, and 102 participants in groups 1, 2, and 3, respectively. We refer to these clusters as the “morning engaged,” “afternoon engaged,” and “unengaged” groups. Figure 1 shows the mean METs for each minute in a day by the three groups after Loess smoothing with span 0.1. The plot in Figure 2 indicates that the morning engaged group engaged in activity earlier than the afternoon engaged group and both groups had similar overall activity level, whereas the unengaged group did not engage in activity as much as the other two groups.

**Table 1:** Comparison of sociodemographic and clinical characteristics among the three clustered groups based on raw metabolic equivalent of tasks (METs) data (N=215).

Sociodemographics and clinical characteristics			Afternoon engaged group (n=65)		Morning engaged group (n=48)		Unengaged group (n=102)		Overall *P* value^a^	
**Sociodemographics**										
	Age (years), mean (SD)		52.2 (11.3)		53.7 (9.8)		51.8 (11.8)		.64	
	**Education, n (%)**								.009^b^	
		High school/some college		21 (32.3)		19 (39.6)		18 (17.6)		
		College/Graduate school		44 (67.7)		29 (60.4)		84 (82.4)		
	**Race, n (%)**								.15	
		White		32 (49.2)		22 (45.8)		63 (61.8)		
		Asian and Pacific Islander		19 (29.2)		15 (31.3)		16 (15.7)		
		Nonwhite and Multiracial		14 (21.5)		11 (22.9)		23 (22.5)		
	Marital status (married/cohabitating), n (%)		39 (60.0)		25 (52.1)		46 (45.1)		.17	
	**Occupation, n (%)**								.19	
		Paid full time/part time		42 (64.6)		38 (79.2)		76 (74.5)		
		Homemaker/retried/disabled		23 (35.4)		10 (20.8)		26 (25.5)		
	Previous pedometer usage, n (%)		34 (52.3)		24 (50.0)		48 (47.1)		.80	
	Drives a car at least once a week, n (%)		57 (87.7)		37 (77.1)		79 (77.5)		.21	
	Has a dog, n (%)		12 (18.5)		12 (25.0)		16 (15.7)		.39	
	Participated in diet plan prior to the study, n (%)		38 (58.5)		25 (52.1)		60 (58.8)		.72	
	Has a gym membership, n (%)		14 (21.5)		11 (22.9)		38 (37.3)		.05	
**Accelerometer (objective measure), mean (SD)**										
	Weekly total minutes of MVPA^c^ by accelerometer with 1 minute criteria		372.1 (137.5)		401.5 (132.8)		205.7 (92.6)		<.001^d^	
	Weekly total minutes of MVPA by accelerometer with 5 minutes criteria		93.2 (90.8)		119.8 (113.1)		63.3(62.1)		.001^e^	
	Weekly total minutes of MVPA by accelerometer with 10 minutes criteria (a 1 or 2 minutes interruption allows)		57.3 (73.3)		78.1 (105.1)		37.8 (47.8)		.006^f^	
	Average daily steps		6436.1 (2216.9)		6722.9 (1718.9)		4796.9 (1723.9)		<.001^g^	
**Other self-reported measures, mean (SD)**										
	Weekly total hours of TV watching and computer usage time		25.5 (17.6)		23.3 (16.8)		30.3 (18.3)		.048	
	**SF-12**^h^									
		Physical Component score	52.0 (6.0)		51.7 (6.1)		50.9 (6.6)		.53	
		Mental Component score	49.2 (8.6)		50.5 (9.6)		46.6 (10.3)		.04	
	Total CESD^i^ score		7.5 (7.1)		8.1 (6.5)		11.9 (8.9)		<.001^j^	
	Total self-efficacy for physical activity score		18.3 (4.4)		19.6 (4.2)		19.1 (5.0)		.30	
	**Social support for physical activity**									
		Total family score	31.1 (9.2)		32 (7.7)		30.9 (10.2)		.82	
		Total friends score	30.8 (7.5)		30.9 (8.1)		32.3 (9.1)		.46	
	Total barriers to being active score		24.0 (9.5)		22.2 (10.0)		23.6 (10.2)		.91	
**Clinical data, mean (SD)**										
	Body mass index (kg/m^2^)		29.3 (6.5)		28.1 (5.3)		29.9 (6.1)		.23	
	Waist circumference (cm)		96.4 (14.4)		94.4 (13.0)		99.0 (14.8)		.17	
	Hip circumference (cm)		109.7 (13.5)		107.2 (12.6)		112.3 (13.9)		.09	
	Resting systolic blood pressure (mm Hg)		121.1 (14.0)		118.8 (13.4)		121.5 (14.7)		.56	
	Resting diastolic blood pressure (mm Hg)		76.1 (10.1)		74.7 (8.9)		78.5 (9.9)		.07	
	Cholesterol, total (mg/dL)		205.5 (35.2)		199.9 (41.4)		203.4 (40.9)		.76	
	Triglycerides (mg/dL)		117.6 (54.3)		103.9 (46.9)		117.0 (53.4)		.31	
	LDL^k^ (mg/dL)		119.5 (33.0)		115.4 (35.7)		118.8 (33.8)		.80	
	HDL^l^ (mg/dL)		62.6 (15.8)		63.7(18.8)		61.2 (16.5)		.68	
	HbA_1c_ (%)		5.8 (0.56)		5.7 (0.4)		5.7 (0.5)		.34	
	Fasting plasma glucose (mg/dL)		95.1 (17.1)		95.5 (12.2)		97.0 (16.0)		.71	

^a^If overall *P* values were significant, pairwise comparisons were made. Only significant pairwise comparison *P* values are reported.

^b^Pairwise comparison between Morning engaged and Unengaged in those with High school/some college: *P*=.008.

^c^MVPA: moderate-to-vigorous physical activity.

^d^Pairwise comparison between Afternoon engaged and Unengaged: *P*<.001; between Morning engaged and Unengaged: *P*<.001.

^e^Pairwise comparison between Morning engaged and Unengaged: *P*=.001.

^f^Pairwise comparison between Between Morning engaged and Unengaged: *P*=.005

^g^Pairwise comparison between Afternoon engaged and Unengaged: *P*=.001; between Morning engaged and Unengaged: *P*<.001.

^h^SF-12: 12-item Short-Form Health Survey.

^i^CES-D: Center for Epidemiological Studies Depression Scale.

^j^Pairwise comparison between Afternoon engaged and Morning engaged: *P*=.001; between Morning engaged and Unengaged: *P*=.02.

^k^LDL: low-density lipoprotein.

^l^HDL: high-density lipoprotein.

In Table 1, sociodemographic self-reported questionnaires and physical activity measures were compared among the three groups. The unengaged group represented 47.4% of the sample (102/215). The unengaged group had significantly lower weekly total minutes of accelerometer-measured MVPA with 10 minutes criteria and mean daily steps than the other two groups (overall *P*=.006 and *P*<.001, respectively). Furthermore, the unengaged group had the highest depressive symptoms score compared with the afternoon engaged and the morning engaged groups (overall *P* value <.001).

### Clustering on Normalized METs ≥3 Data

**Figure 3:**
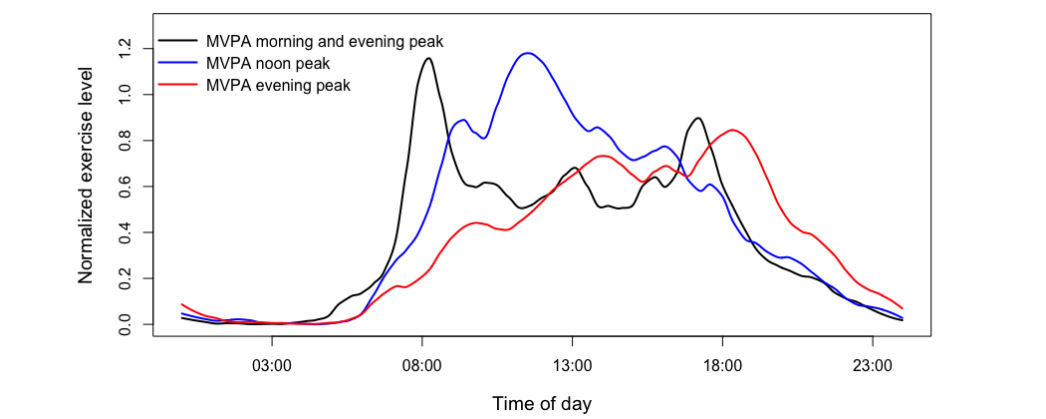
A k-means cluster analysis of normalized METs (metabolic equivalent of tasks) ≥3 data (N=215). MVPA: moderate-to-vigorous physical activity.

The k-mean clustering separated the participants into three groups (Figure 3). This number of clusters was also chosen by the elbow method, and it showed the within-group sum of squares corresponding to the different number of clusters, and using four clusters did not reduce the within-group sum of squares significantly (Multimedia Appendix 2). There were 46, 61, and 108 participants in groups 1, 2, and 3, respectively. We will refer to these groups as the MVPA morning and evening active, MVPA noon peak active, and MVPA evening peak active groups. The clusters were named as such because the MVPA morning and evening active group engaged in MVPA either in the morning or in the evening, the MVPA noon peak active group engaged in MVPA around noon, and the MVPA evening peak active group engaged in MVPA in the evening and at night. The mean normalized METs for each group are shown in Figure 3 after Loess smoothing with span 0.1. We can interpret the vertical axis as the frequency of participants in that group who engaged in MVPA at a particular time. The MVPA morning and evening active group had two peaks: one in the morning and one in the evening. The MVPA noon peak active group tended to engage in MVPA around noon and did slightly less in the evening. The evening peak active group tended to gradually increase MVPA toward evening and with a peak around 6 pm. As seen in Table 2, the MVPA evening peak group (n=108) had higher BMI (*P*=.03), waist circumference (*P*=.02), and hip circumference (*P*=.03) than the MVPA noon peak group (n=61).

**Table 2:** Comparison of sociodemographic and clinical characteristics among three clustered groups based on normalized METs ≥3 data (N=215).

Sociodemographic and clinical characteristics			MVPA^a^ Morning and evening peak group (n=46)		MVPA Noon peak group (n=61)		MVPA Evening peak group (n=108)		Overall *P* value^b^	
**Sociodemographics**										
	Age (years), mean (SD)			51.9 (11.7)		52.4 (10.5)		52.6 (11.5)		.94
	**Education, n (%)**									.81
		High school/some college		11 (23.9)		18 (29.5)		29 (26.9)		
		College/Graduate school		35 (76.1)		43 (70.5)		79 (73.1)		
	**Race, n (%)**									.20
		White		20 (43.5)		31 (50.8)		66 (61.1)		
		Asian and Pacific Islander		12 (26.1)		18 (29.5)		20 (18.5)		
		Nonwhite and multiracial		14 (30.4)		12 (19.7)		22 (20.4)		
	Marital status (Married/cohabitating), n (%)			23 (50.0)		36 (59.0)		51 (47.2)		.33
	**Occupation, n (%)**									.11
		Paid full time/part time		39 (84.8)		43 (70.5)		74 (68.5)		
		Homemaker/retried/disabled		7 (15.2)		18 (29.5)		34 (31.5)		
	Previous pedometer usage, n (%)			25 (54.3)		29 (47.5)		52 (48.1)		.74
	Drives a car at least once a week, n (%)			38 (82.6)		48 (78.7)		87 (80.6)		.88
	Has a dog, n (%)			9 (19.6)		13 (21.3)		18 (16.7)		.74
	Participated in diet plan prior to the study, n (%)			26 (56.5)		31 (50.8)		66 (61.1)		.43
	Has a gym membership, n (%)			18 (39.1)		13 (21.3)		32 (29.6)		.13
**Accelerometer (objective measure), mean (SD)**										
	Weekly total minutes of MVPA by accelerometer with 1 minute criteria			287.7 (121.1)		388.4 (168.9)		254.7 (121.2)		<.001^c^
	Weekly total minutes of MVPA by accelerometer with 5 minutes criteria			70.7 (65.0)		120.1 (113.2)		71.1 (72.6)		.001^d^
	Weekly total minutes of MVPA by accelerometer with 10 minutes criteria (a 1-or 2 minutes interruption allows)			41.0 (48.8)		81.3 (101.8)		41.5 (57.2)		.001^e^
	Average daily steps			5754.0 (1560.4)		6663.7 (2359.9)		5211.7 (1936.8)		<.001^f^
**Other self-reported measures, mean (SD)**										
	Weekly total hours of TV watching and computer usage time			28.7 (19.5)		27.7 (19.5)		26.5 (16.4)		.76
	**SF-12**^g^									
		Physical Component score		52.3 (5.9)		50.9 (7.1)		51.3 (6.0)		.51
		Mental Component score		49.3 (7.6)		47.7 (11.4)		48.1 (9.5)		.69
	Total CES-D^h^ score		9.5 (8.6)		9.9 (8.3)		9.8 (7.9)		.97	
	Total self-efficacy for physical activity score		20.0 (4.5)		18.5 (4.3)		18.8 (4.8)		.23	
	**Social support for physical activity**									
		Total family score		31.1 (8.2)		32.6 (8.8)		30.5 (10.1)		.36
		Total friends score		29.8 (7.8)		31.3 (7.7)		32.5 (8.9)		.20
	Total barriers to being active score		21.3 (10.0)		25.6 (9.1)		23.5 (10.2)		.08	
**Clinical data, mean (SD)**										
	Body mass index (kg/m^2^)			29.6 (6.1)		27.6 (5.5)		30.2 (6.2)		.03^i^
	Waist circumference (cm)			95.9 (14.2)		93.6 (12.8)		99.7 (14.9)		.02^j^
	Hip circumference (cm)			111.3 (13.3)		106.5 (12.6)		112.2 (13.9)		.03^k^
	Resting systolic blood pressure (mm Hg)			120.0 (14.1)		119.4 (13.7)		121.7 (14.6)		.57
	Resting diastolic blood pressure (mm Hg)			75.3 (10.7)		75.2 (8.7)		78.6 (9.9)		.04
	Cholesterol, total (mg/dL)			198.6 (41.0)		203.9 (32.8)		204.9 (41.8)		.66
	Triglycerides (mg/dL)			110.4 (54.9)		117.8 (54.8)		113.9 (50.2)		.77
	LDL^l^ (mg/dL)			113.7 (36.1)		118.4 (28.6)		120.1 (35.7)		.57
	HDL^m^ (mg/dL)			62.9 (18.1)		62.0 (15.6)		62.0 (17.0)		.95
	HbA_1c_ (%)			5.8 (0.5)		5.8 (0.6)		5.7 (0.5)		.68
	Fasting plasma glucose (mg/dL)			95.3 (12.3)		98.2 (17.2)		95.2 (15.8)		.46

^a^MVPA: moderate-to-vigorous physical activity.

^b^If overall *P* values were significant, pairwise comparisons were made. Only significant pairwise comparison *P* values are reported.

^c^Pairwise comparison between Morning and evening peak and Noon peak: *P*=.001; between Noon peak and Evening peak: *P*<.001.

^d^Pairwise comparison between Morning and evening peak and Noon peak: *P*=.009; between Noon peak and Evening peak: *P*<.001.

^e^Pairwise comparison between Morning and evening peak and Noon peak: *P*=.01; between Noon peak and Evening peak: *P*=.002.

^f^Pairwise comparison between Noon peak and Evening peak: *P*<.001.

^g^SF-12: 12-item Short-Form Health Survey.

^h^CES-D: Center for Epidemiological Studies Depression Scale.

^i^Pairwise comparison between Noon peak and Evening peak: *P*=.03.

^j^Pairwise comparison between Noon peak and Evening peak: *P*=.02.

^k^Pairwise comparison between Noon peak and Evening peak: *P*=.02.

^l^LDL: low-density lipoprotein.

^m^HDL: high-density lipoprotein.

## Discussion

### Principal Results

This study is the first to identify clusters of women aged between 25 and 69 years based on seven consecutive days of accelerometer-measured METs and MVPA (≥3 METs). This first cluster analysis successfully identified three groups based on accelerometer-measured METs. It appears that only the difference between the afternoon engaged and the morning engaged groups is timing of activity throughout the day. However, the unengaged group (representing 47.4% of the sample) had a much lower activity level than the other two groups.

We found that the unengaged group was more likely to have a college or graduate degree compared to the afternoon engaged and morning engaged groups. In the cluster analysis study of self-reported physical activity involving 3324 individuals in France, Omorou et al [32] also reported that individuals with high socioeconomic status were less likely to engage in occupational physical activity and active transportation compared to those with low socioeconomic status. In contrast, there is an inverse association between leisure physical activity and socioeconomic status [33]. In other words, individuals with high socioeconomic status had greater leisure physical activity time than those with low socioeconomic status.

Furthermore, consistent with previous study findings [34,35], the unengaged group had a significantly higher depressive symptom score than the other two groups. It is estimated that approximately 20% to 25% of female adults have significantly elevated depressive symptoms (eg, CES-D score ≥16 points) [36]. This inverse association between depressive symptoms and physical activity levels has been well established. More than a dozen RCTs have examined the effect of physical activity on depressive symptoms, and some studies demonstrated physical activity could prevent or mitigate mild-to-moderate depressive symptoms [37]. The unengaged group may respond to a physical activity intervention differently compared with the afternoon active and morning active groups. However, this assumption needs to be confirmed in further studies.

The second cluster analysis based on MVPA (normalized 3 ≥METs data) also showed three distinct groups (MVPA morning and evening peak, MVPA noon peak, and MVPA evening peak). A two-peak pattern of MVPA (7-8 am and 5-6 pm) in the MVPA morning and evening peak group might be explained by active commuting. The MVPA noon peak group appeared to have the greatest duration of MVPA compared with the other two groups. Moreover, this MVPA noon peak group had significantly lower metabolic risks (BMI, hip and waist circumferences) than the MVPA evening peak group. In a recent large epidemiologic study, the investigators also reported that bouts of 10 minutes or more of MVPA (as per current guidelines) and even bouts of less than 10 minutes were associated with lower levels of adiposity and a lower risk of metabolic syndrome in older adults [38]. The other studies found that bouts of at least 10 minutes of MVPA were a stronger predictor for metabolic risks than bouts of less than10 minutes of MVPA [39,40]. Given the benefit of MVPA regardless of its duration, less emphasis on bouts of at least 10 minutes of MVPA might help encourage physically inactive women to engage in MVPA throughout the day.

### Strengths and Limitations of the Study

The strengths of this study were that we were able to use seven consecutive days of accelerometer-measured physical activity data instead of depending on participant recall to collect the vast majority of types of activities (active transportation, occupational and leisure activity), and to identify physical activity patterns that were specific to certain times of the day. In addition, the participants were not able to view their steps taken and intensity of physical activity during the data collection period; thus, this blinding function helped prevent participants from modifying their daily activity. Despite these strengths, some limitations need to be taken into account. First, the findings of this study might not be generalizable to men or children. Men tend to be more active than women are across their life spans. Second, in general, individuals with high levels of depressive symptoms are less likely to be enrolled in clinical studies compared to those with low symptoms. The proportion of the unengaged group could be larger than this data. Lastly, the accelerometer used in the mPED trial was not able to capture activities such as swimming, bicycling, and weight lifting. However, women who engaged in these activities in the mPED trial were relatively low in this sample [7].

Despite the use of objectively measured physical activity, the sample size was relatively small in this study. Thus, these identified cluster groups need to be cross-validated using a large national dataset such as the National Health and Nutrition Examination Survey.

### Conclusions

Classifying physically inactive individuals into more precise activity patterns could assist in tailoring the timing, frequency, duration, and intensity of physical activity interventions for women. For example, recommending bouts of physical activity before noon to the unengaged group or MVPA evening peak group may lead to an increase in their activity levels. Future research should consider examining how different types of baseline physical activity cluster groups will respond to different types of physical activity interventions.

## Appendix

Multimedia Appendix 1 Result of the Elbow Method for the raw METs data.

Multimedia Appendix 2 Result of the Elbow Method for the normalized METs ≥3 data.

## Abbreviations

BMI: body mass index
CES-D: Center for Epidemiological Studies Depression Scale
HDL: high-density lipoprotein
LDL: low-density lipoprotein
MET: metabolic equivalent of task
mPED: Mobile Phone Based Physical Activity Education
MVPA: moderate-to-vigorous physical activity
RCT: randomized controlled trial
SF-12: 12-item Short-Form Health Survey
