# Supplementary figures and images for: Objectively Measured Baseline Physical Activity Patterns in Women in the mPED Trial: Cluster Analysis
Source: JMIR Public Health Surveill. 2018 Feb 1;4(1):e10. doi: 10.2196/publichealth.9138 (PMC5814604; doi:10.2196/publichealth.9138)

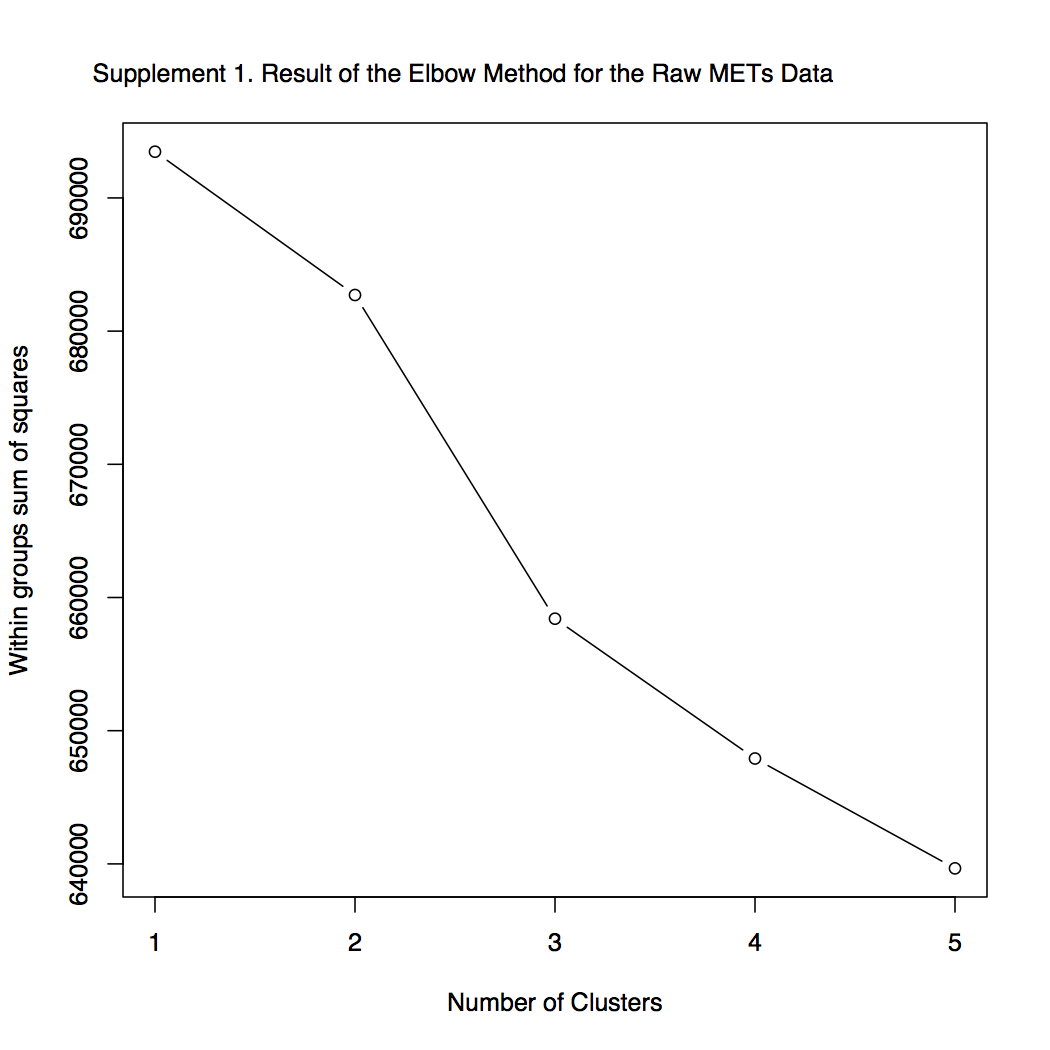

Supplement: Multimedia Appendix 1 [file publichealth_v4i1e10_app1.png]

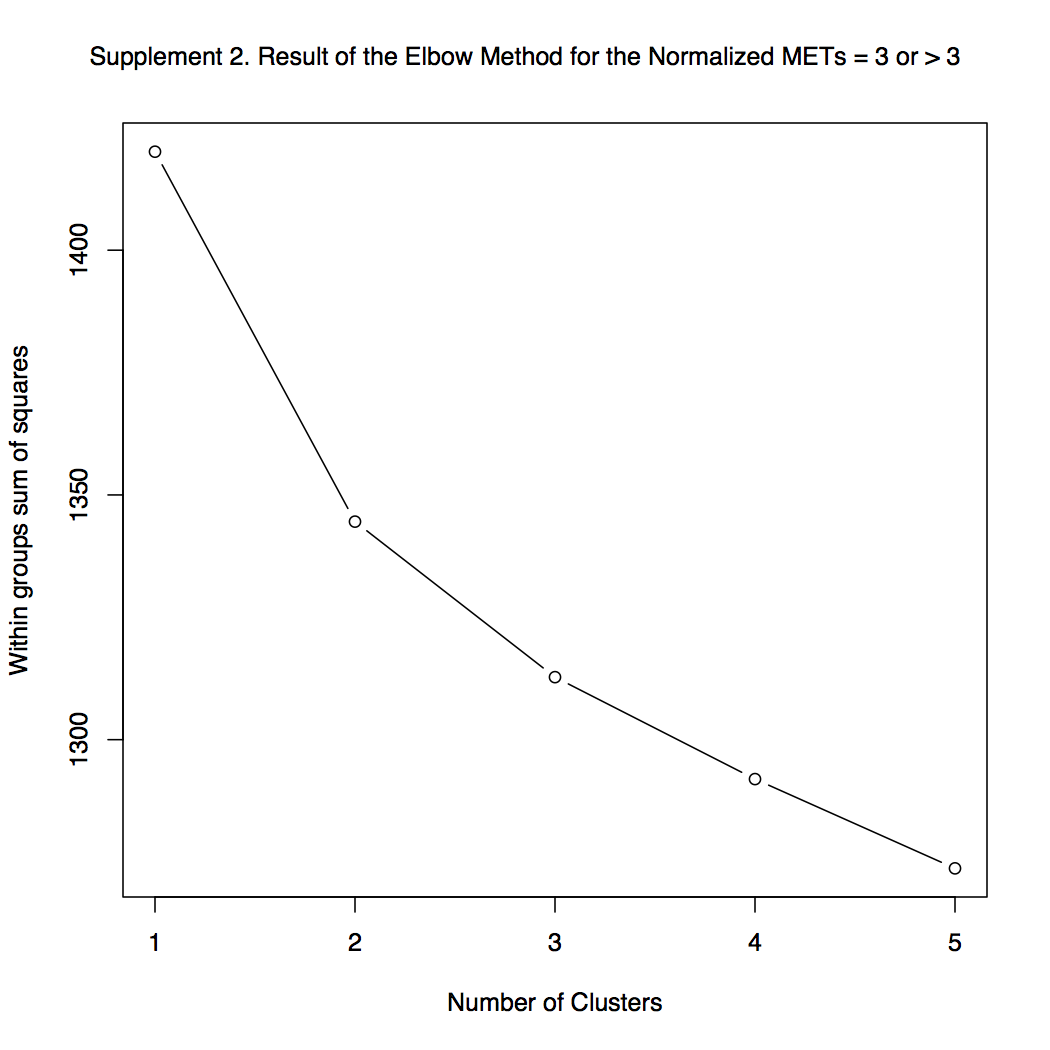

Supplement: Multimedia Appendix 2 [file publichealth_v4i1e10_app2.png]
